# Effect of Quinolone Prophylaxis Discontinuation During Pre-engraftment Neutropenia on Incidence, Mortality, and Etiology of Bloodstream Infections in Hematopoietic Stem-cell Transplant Recipients: A Systematic Review and Meta-analysis

**DOI:** 10.1093/ofid/ofag358

**Published:** 2026-06-08

**Authors:** Bruno Azevedo Randi, Hermes Ryoiti Higashino, Patrick Leon de Godoy Macedo, Thaís Guimarães, Bruno Garcia Pires, Vanderson Rocha, Alex Howard, Anna Sara Levin, Silvia Figueiredo Costa

**Affiliations:** Departamento de Infectologia e Medicina Tropical, Hospital das Clínicas HCFMUSP, Faculdade de Medicina da Universidade de São Paulo, São Paulo, Brazil; Departamento de Infectologia e Medicina Tropical, Hospital das Clínicas HCFMUSP, Faculdade de Medicina da Universidade de São Paulo, São Paulo, Brazil; Departamento de Infectologia e Medicina Tropical, Hospital das Clínicas HCFMUSP, Faculdade de Medicina da Universidade de São Paulo, São Paulo, Brazil; Departamento de Controle de Infecção Hospitalar do Hospital das Clínicas HC-FMUSP, Faculdade de Medicina, Universidade de São Paulo, São Paulo, Brazil; Departamento de Hematologia, Hemoterapia e Terapia Celular, Hospital das Clínicas HC-FMUSP, Faculdade de Medicina, Universidade de São Paulo, São Paulo, Brazil; Departamento de Hematologia, Hemoterapia e Terapia Celular, Hospital das Clínicas HC-FMUSP, Faculdade de Medicina, Universidade de São Paulo, São Paulo, Brazil; Department of Antimicrobial Pharmacodynamics and Therapeutics, Institute of Systems, Molecular and Integrative Biology, University of Liverpool, Liverpool, UK; Department of Infection and Immunity, Liverpool Clinical Laboratories, Liverpool University Hospitals NHS Foundation Trust, Liverpool, UK; Departamento de Infectologia e Medicina Tropical, Hospital das Clínicas HCFMUSP, Faculdade de Medicina da Universidade de São Paulo, São Paulo, Brazil; Departamento de Infectologia e Medicina Tropical, Hospital das Clínicas HCFMUSP, Faculdade de Medicina da Universidade de São Paulo, São Paulo, Brazil; Laboratório de Investigação Médica em Protozoologia, Bacteriologia e Resistência Antimicrobiana - LIM/49, Faculdade de Medicina FMUSP, Universidade de São Paulo, São Paulo, Brazil; Centres for Antimicrobial Optimisation Network, CAMO-Net, Brazil

**Keywords:** hematopoietic stem-cell transplantation, prophylaxis, quinolone

## Abstract

**Background:**

Bloodstream infections (BSIs) are a major complication during pre-engraftment neutropenia in hematopoietic stem-cell transplant (HSCT) recipients. Quinolone prophylaxis reduces BSIs but increases multidrug-resistance bacterial infections. We aimed to assess the impact of discontinuing quinolone prophylaxis in adult HSCT recipients.

**Methods:**

We performed a systematic review with meta-analysis. Five databases were searched on May 20, 2025. Studies were eligible for inclusion if they reported clinical characteristics of adult HSCT recipients and outcomes following the discontinuation of prophylaxis. Data extraction and quality assessment were performed independently by 2 reviewers. Meta-analysis was performed and pooled risk ratios with 95% CI calculated using a random-effects model.

**Results:**

Ten studies including 2363 HSCT recipients (1190 with and 1173 without prophylaxis) were included. Most were allogeneic-HSCT (53.1%). Prophylaxis was associated with a lower risk of BSIs due to any microorganism (pooled risk ratio: 0.69; 95% CI 0.54–0.87; *P* value = .002; I^2^ = 61%) and due to Gram-negative bacteria (pooled risk ratio: 0.49; 95% CI 0.33–0.74; *P* value = .0007; I^2^ = 71%). In the overall population, there was no significant difference in resistant infections. In patients with Gram-negative BSIs, prophylaxis was associated with a higher risk of quinolone-resistant (pooled risk ratio: 2.35; 95% CI 1.68–3.29; *P* value < .00001; I^2^ = 27%) and carbapenem-resistant microorganisms (pooled risk ratio: 5.32; 95% CI 1.08–26.16; *P* value = .04; I^2^ = 69%). Discontinuation was not associated with a statistically increased infection-related mortality (pooled risk ratio: 1.26; 95% CI: 0.71–2.23; *P* value = .43; I^2^ = 0%).

**Conclusions:**

Discontinuation of quinolone prophylaxis was associated with an elevated incidence of BSIs, without a concomitant reduction in the overall burden of antimicrobial resistance. Importantly, infection-related mortality did not increase to a statistically significant extent. Furthermore, Gram-negative BSIs occurring after discontinuation were less likely to exhibit resistance to quinolones or carbapenems.

## Teaser

Our systematic review with meta-analysis suggests that discontinuation of quinolone prophylaxis during pre-engraftment neutropenia reduces bacterial resistance in Gram-negative BSI. No statistically significant difference in infection-related mortality was observed. These findings may support reconsideration of this strategy in HSCT centers.

Bloodstream infections (BSIs) are a major infectious complication during pre-engraftment neutropenia in hematopoietic stem-cell transplant (HSCT) recipients [1, 2]. In this phase, the risks for BSIs are related to the presence of long-term venous catheters, mucositis and prolonged neutropenia [1].

Quinolone prophylaxis during neutropenia in cancer patients, including during pre-engraftment neutropenia in HSCT recipients, has been shown to be effective in preventing BSIs, as demonstrated in a previous randomized clinical trial [3]. Subsequently, a Cochrane systematic review evidenced that quinolone prophylaxis was associated with reduced all-cause mortality in neutropenic cancer patients [4]. Based on this evidence, clinical guidelines have since recommended the use of quinolone prophylaxis in these patients, including those undergoing HSCT [5].

However, following the implementation of this strategy, infections caused by multidrug-resistant bacteria have increased, and some studies have begun to question this strategy [6, 7]. Given this scenario, some centers have evaluated the discontinuation of routine quinolone prophylaxis in the HSCT setting [8, 9].

Thus, the aim of this study was to analyze all studies that evaluated the impact of discontinuing quinolone prophylaxis during pre-engraftment neutropenia in adult HSCT recipients by performing a systematic review and meta-analysis.

## METHODS

### Search Strategy

This review was conducted in accordance with the Preferred Reporting Items for Systematic Reviews and Meta-Analyses guidelines [10]. The following 5 databases were searched on October 10, 2023, and updated on May 20, 2025: MedLine, Embase, SCOPUS, LILACS, and Web of Science. No date or language restrictions were applied to the search strategy.

Supplementary Appendix 1 describes in detail the search strategies. The protocol has been registered with PROSPERO (CRD42023467803), The International Prospective Register of Systematic Reviews.

### Inclusion and Exclusion Criteria

Two reviewers (B.A.R. and P.L.G.M.) independently screened the papers’ titles and abstracts for inclusion. The same inclusion criteria were applied to the assessment of full-text articles. Any discrepancies were resolved through discussion and consensus between the 2 reviewers, or by arbitration of a third reviewer (H.R.H. or S.F.C.) if necessary.

Studies in any language were screened for eligibility. Studies were eligible for inclusion if they met the following criteria: (1) reported clinical characteristics of adult HSCT recipients (aged 18 years old or above); (2) evaluated outcomes following the discontinuation of quinolone prophylaxis as a change in institutional practice over time (ie, before–after designs); and (3) were full-text articles. Studies comparing contemporaneous groups receiving versus not receiving quinolone prophylaxis, without a defined discontinuation strategy over time, were excluded. In addition, studies enrolling mixed populations (eg, adults and children or HSCT and non-HSCT patients) were only eligible if outcomes were reported separately for the relevant subgroups; otherwise, they were excluded. Searches were not limited by any date or language restriction.

### Data Extraction

Data from included articles were independently extracted by 2 reviewers (B.A.R. and P.L.G.M.), using a form designed specifically for this review. In the event of discrepancies, they consulted a third reviewer (H.R.H. or S.F.C.) for a final decision. The following data were extracted from the included articles:

- General information: year of publication, study design, and country of origin.
- Transplant and patient`s characteristics: number of patients included, age, gender, type of transplant (autologous or allogeneic), main underlying diseases, follow-up duration, and type of quinolone used (levofloxacin or ciprofloxacin).
- Outcomes: number of BSI before and after discontinuation of quinolone prophylaxis; number of infections caused by quinolone and carbapenem-resistant organisms before and after discontinuation; prevalence of *Clostridioides difficile* infection (CDI) before and after discontinuation; infection-related mortality during follow-up, stratified by quinolone prophylaxis status. Mortality time points were extracted as reported in each study. Overall mortality was not used as an outcome, as it includes causes of death not directly preventable by antibacterial prophylaxis (eg, graft-vs-host disease or relapse-related mortality), which may dilute the effect of the intervention. Infection-related mortality, although not restricted to bacterial causes and potentially including viral or fungal infections, was considered a more specific outcome and more closely related to infectious complications.

Age was reported as mean and standard deviation (SD). When possible, studies that presented age using other measures (ie, median with range or interquartile range), were converted using formulas previously described by Hozo et al [11] and Wan et al [12].

### Quality Evolution

To assess the methodological quality of the included studies, we used the Joanna Briggs Institute (JBI) Critical Appraisal Checklist for Quasi-experimental Studies [13].

The checklist comprises 9 items. Each item was rated as ‘yes,’ no,’ or ‘unclear.’ Studies meeting 8–9 criteria were classified as low risk of bias, those meeting 6–7 criteria as moderate risk, and those meeting ≤6 criteria as high risk of bias, as previously applied in similar systematic reviews.

### Statistical Analysis

We conducted the meta-analysis using Review Manager (RevMan), version 5.4.1. Pooled risk ratios with 95% confidence intervals (95% CI) were calculated using a random-effects model for the following outcomes: BSIs due to any microorganism and Gram-negative bacteria; infections due to quinolone-resistant microorganisms (in the overall population and among patients with Gram-negative BSI) and carbapenem-resistant Gram-negative bacteria (among patients who developed Gram-negative BSI); CDI; and infection-related mortality during follow-up. Subgroup analyses were performed, when feasible, according to the type of quinolone used (levofloxacin vs ciprofloxacin) for outcomes with sufficient data.

The I^2^ statistic was used to measure heterogeneity. Funnel plots were generated only for outcomes that included 10 studies.

## RESULTS

### Study Selection

**Figure 1.**
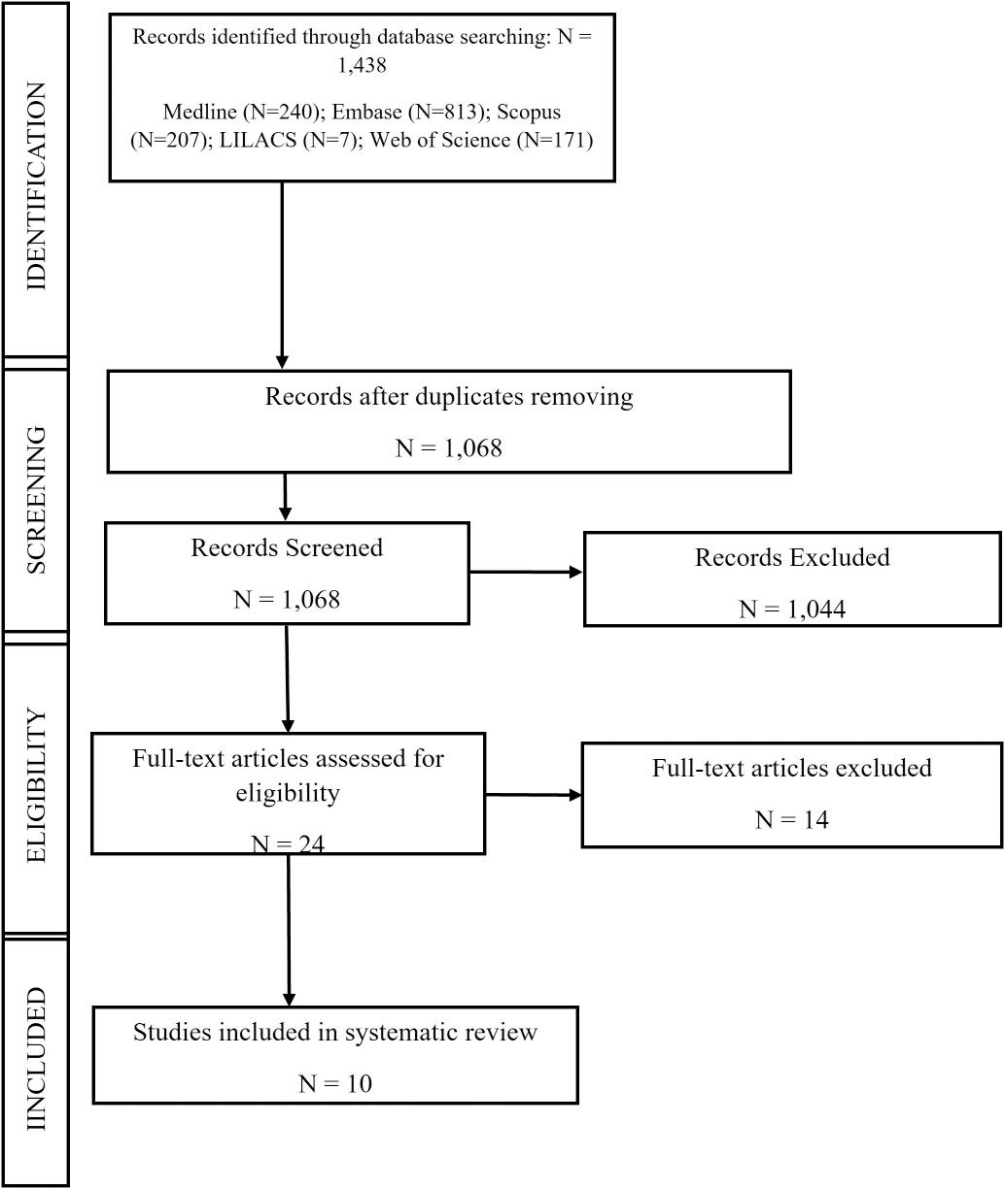
Flowchart of the selection of the studies included in the systematic review.

We identified 1438 studies that met the selection criteria, 1068 nonduplicate titles were screened for eligibility and 24 were retrieved for full-text evaluation. Fourteen were excluded because they did not evaluate the discontinuation of quinolone prophylaxis in centers where this strategy had already been implemented (ie, older studies that assessed the introduction of antibacterial prophylaxis), or studies including both adults and children without stratified results. Thus, 10 studies were included in the analysis (Figure 1) [8, 9, 14–21].

### Included Studies Main Characteristics

The main characteristics of the included studies are summarized in Table 1. All studies were published in English, except Sojo et al [16], which was published in Spanish. The first study to evaluate the impact of discontinuing quinolone prophylaxis in HSCT recipients was conducted in Japan and published in 2010 [14].

**Table 1.** Characteristics of the Studies Included in the Systematic Review: Effect of Quinolone Prophylaxis Discontinuation in HSCT Patients

Reference, Year	Country	Study Design	Study Period	Total Time of Study (mo)	Quinolone	Total Time Without Prophylaxis (mo)	No of HSCT Recipients
Kanda J, 2010	Japan	Quasi-experimental	2000–2008	108	Levofloxacin	52	128
Sohn BS, 2012	South Korea	Quasi-experimental	2001–2008	90	Ciprofloxacin	52	232
Sojo JF, 2016	Spain	Quasi-experimental	2006–2010	49	Levofloxacin	24	239
Yeshurun M, 2018	Israel	Quasi-experimental	2007–2016	118	Ciprofloxacin	68	356
Daoud-Asfour H, 2022	Israel	Quasi-experimental	2018–2020	29	Ciprofloxacin	18	129
Guimarães T, 2022	Brazil	Quasi-experimental	2016–2019	48	Levofloxacin	12	310
Clerici D, 2022	Italy	Quasi-experimental	2018–2020	36	Levofloxacin	22	359
Nair A, 2023	India	Quasi-experimental	2012–2021	120	Levofloxacin	60	135
Stern A, 2024	Israel	Quasi -experimental	2017–2020	48	Ciprofloxacin	24	254
Neuerburg CKF, 2024	Germany	Quasi -experimental	2012–2020	91	Ciprofloxacin	31	221

Abbreviations: Mo, month; NA, not available.

Six studies (60.0%) were conducted in Asia: Israel (3), India (1), Japan (1), and South Korea (1). Three studies (30.0%) were from Europe: Spain (1), Italy (1), and Germany (1). One study (10.0%) was conducted in South America: Brazil.

All included studies were quasi-experimental and unicentric [8, 9, 14–21]. Study durations varied from 29 [18] to 120 [19] months. The period without quinolone prophylaxis varied from 12 [8] to 68 [17] months. Five studies (50.0%) [8, 9, 14, 16, 19] used levofloxacin for prophylaxis, while another 5 [15, 17, 18, 20, 21].

### Quality Evolution

Using the JBI checklist, we identified 6 (60.0%) studies with moderate risk of bias and 4 (40.0%) with low risk of bias. The results of the assessment are described in Supplementary Appendix 2.

### Patient´s Baseline Characteristics

A total of 2363 HSCT recipients were included. Table 2 summarizes the main baseline characteristics of the patients. Most were allogeneic-HSCT patients (n = 1,257, 53.1%). The mean age ranged from 41.2 ± 8.5 [14] to 59.0 ± 10.3 [9] years old. In all the studies, male sex was more prevalent (n = 1383, 58.5%).

**Table 2.** Main Underline patients’ Characteristics: Effect of Quinolone Prophylaxis Discontinuation in HSCT Patients

Reference, Year	Allogeneic N (%)	Autologous N (%)	Age Mean ± SD	Male Sex N (%)	Main Underline Diseases N (%)	Patients Under Prophylaxis N (%)	Patients Without Prophylaxis N (%)	Auto-HSCT Under Prophylaxis N (%)	Auto-HSCT Without Prophylaxis N (%)	Allo-HSCT Under Prophylaxis N (%)	Allo-HSCT Without Prophylaxis N (%)
Kanda J, 2010	128 (100.0)	0	41.2 ± 8.5	69 (53.9)	Myeloid neoplasms: 79 (61.7) Lymphoid neoplasms: 49 (38.2)	87 (67.9)	41 (32.0)	0	0	87 (67.9)	41 (32.0)
Sohn BS, 2012	0	232 (100)	No prophylaxis: 49.1 ± 10.4 Prophylaxis2: 51.4 ± 7.2	137 (59.0)	MM: 153 (65.9) NHL: 80 (34.4)	114 (49.1)	118 (50.8)	114 (49.1)	118 (50.8)	0	0
Sojo JF, 2016	101 (42.2)	138 (57.7)	No prophylaxis: 50.4 ± 12.2 Prophylaxis: 48.8 ± 13.3	142 (59.4)	AL: 75 (31.3) MM: 68 (28.4) NHL: 47 (19.6) HL: 18 (7.5)	132 (55.2)	107 (44.7)	79 (60.0)	59 (55.0)	53 (40.0)	48 (45.0)
Yeshurun M, 2018	0	356 (100.0)	57.4 ± 9.0	194 (59.0)	MM: 202 (56.7) Lymphomas: 154 (43.2)	177 (49.7)	179 (50.2)	177 (49.7)	179 (50.2)	0	0
Daoud-Asfour H, 2022	129 (100.0)	0	53.6 ± 10.4	72 (55.8)	Leukemia: 106 (82.1) Lymphomas: 11 (8.5)	75 (58.1)	54 (41.9)	0	0	75 (58.1)	54 (41.8)
Guimarães T, 2022	66 (21.0)	244 (79.0)	51.9 ± 17.8	167 (54.0)	MM: 107 (35.0) NHL: 50 (16.0) HL: 40 (13.0) Leukemia: 34 (10.9)	222 (72.0)	88 (28.0)	183 (82.0)	61 (69.0)	39 (18.0)	27 (31.0)
Clerici D, 2022	223 (62.1)	136 (37.8)	Autologous: 59.0 ± 10.3 Allogeneic: 53.8 ± 17.9 (No data for the entire population)	232 (64.6)	Lymphoid disorders: 181 (50.4) Myeloid disorders: 168 (46.7)	109 (30.3)	250 (69.6)	38 (27.9)	98 (72.0)	71 (31.8)	152 (68.1)
Nair A, 2023	135 (100.0)	0	No prophylaxis: 26.6 ± 15.8 Prophylaxis: 26.7 ± 13.8	95 (79.1)	Malignant diseases: 99 (73.3) Nonmalignant diseases: 36 (26.6)	43 (31.8)	92 (68.1)	0	0	43 (31.8)	92 (68.1)
Stern A, 2024	254 (100.0)	0	53.2 ± 17.1	149 (58.6)	AL/MDS: 199 (78.3) Lymphoma: 18 (7.1)	130 (51.0)	124 (49.0)	0	0	130 (51.0)	124 (49.0)
Neuerburg CKF, 2024	221 (100.0)	0	56.3 ± 12.0	126 (57.0)	AML/MDS: 135 (61.1) NHL: 31 (14.1) ALL: 20 (9.0)	101 (45.7)	120 (54.2)	0	0	101 (45.7)	120 (54.2)

Abbreviations: AL: acute leukemias; ALL, acute lymphoblastic leukemia; AML, acute myeloid leukemia; HL, Hodgkin lymphoma; MDS, myelodysplastic syndrome; MM, multiple myeloma; NA: not available; NHL, non-Hodgkin lymphoma; SD: standard deviation.

The main underlying hematologic diseases were leukemia/myelodysplastic syndrome (n = 816, 34.5%), lymphomas (n = 679, 28.7%), and multiple myeloma (n = 530, 22.4%).

A total of 1190 HSCT patients (50.3%) were evaluated during quinolone prophylaxis, including 591 (49.6%) autologous and 599 (50.3%) allogeneic transplants. In contrast, 1173 patients (49.6%) were evaluated in the absence of quinolone prophylaxis, with 515 (43.9%) autologous and 658 (56.0%) allogeneic transplants.

### Bloodstream Infections

Table 3 presents the prevalence of BSIs caused by any microorganism and by Gram-negative bacteria. Supplementary Appendix 3 presents the prevalence of blood cultures with Enterobacteriales or nonfermentative Gram-negative bacteria.

**Table 3.** Prevalence of Bloodstream Infection, Antimicrobial Resistance, *Clostridioides Difficile* Infection and Death: Effect of Quinolone Prophylaxis Discontinuation in HSCT Patients

Reference, Year	BSI (any Microorganism)			GN-BSI			Quinolone-resistant Microorganisms			Carbapenem-resistant GN			CDI			Infection-related Mortality		
	Prophylaxis N (%)	No prophylaxis N (%)	*P* Value	Prophylaxis N (%)	No prophylaxis N (%)	*P* Value	Prophylaxis N (%)	No prophylaxis N (%)	*P* Value	Prophylaxis N (%)	No prophylaxis N (%)	*P* Value	Prophylaxis N (%)	No prophylaxis N (%)	*P* Value	Prophylaxis N (%)	No prophylaxis N (%)	*P* Value
Kanda J, 2010	6 (7.0)	6 (15.0)	.45	2 (33.3)	4 (66.6)	NA	6 (100.0)	2 (33.3)	NA	NA	NA	NA	NA	NA	NA	NA	NA	NA
Sohn BS, 2012	14 (12.2)	16 (13.5)	NA	5 (35.7)	5 (31.2)	.84	4 (3.5)	0	.04	0	0	NA	11 (10.0)	20 (8.0)	NA	1 (0.8)	0	NA
Sojo JF, 2016	54 (41.0)	43 (40.0)	.26	15 (11.0)	41 (38.0)	.001	52 (39.0)	15 (14.0)	<.001	NA	NA	NA	NA	NA	NA	NA	NA	NA
Yeshurun M, 2018	8 (4.5)	27 (15.0)	<.0001	6 (3.3)	20 (11.2)	.003	5 (62.5)	5 (18.5)	.01	NA	NA	NA	5 (2.8)	12 (6.7)	.008	NA	NA	NA
Daoud-Asfour H, 2022	26 (34.7)	22 (40.7)	.60	22 (29.3)	15 (27.8)	1.00	NA	NA	NA	NA	NA	NA	NA	NA	NA	NA	NA	NA
Guimarães T, 2022	30 (14.0)	30 (34.0)	<.001	23 (76.6)	24 (80.0)	NA	18 (60.0)	5 (17.0)	.001	7 (23.0)	3 (10.0)	.30	NA	NA	NA	8 (3.6)	1 (1.1)	.03
Clerici D, 2022	28 (25.6)	101 (40.4)	NA	10 (9.1)	71 (28.4)	NA	9 (90.0)	32 (45.0)	.03	5 (50.0)	3 (4.22)	.001	NA	NA	NA	6 (5.5)	15 (6.0)	NA
Nair A, 2023	16 (37.0)	31 (34.0)	.70	16 (37.0)	31 (34.0)	.70	NA	NA	NA	NA	NA	NA	NA	NA	NA	8 (18.6)	13 (14.1)	NA
Stern A, 2024	33 (25.4)	50 (40.3)	NA	24 (21.0)	58 (33.0)	.02	14 (68.9)	18 (41.6)	.02	NA	NA	NA	5 (3.8)	14 (11.3)	.04	NA	NA	NA
Neuerburg CKF, 2024	21 (20.8)	36 (30.8)	.16	1 (1.0)	10 (8.3)	.02	NA	NA	NA	NA	NA	NA	23 (22.8)	3 (2.5)	<.001	NA	NA	NA

Abbreviations: BSI, bloodstream infection; CDI, *Clostridioides difficile* infection; GN, Gram-negative; NA, not available.

**Figure 2.**
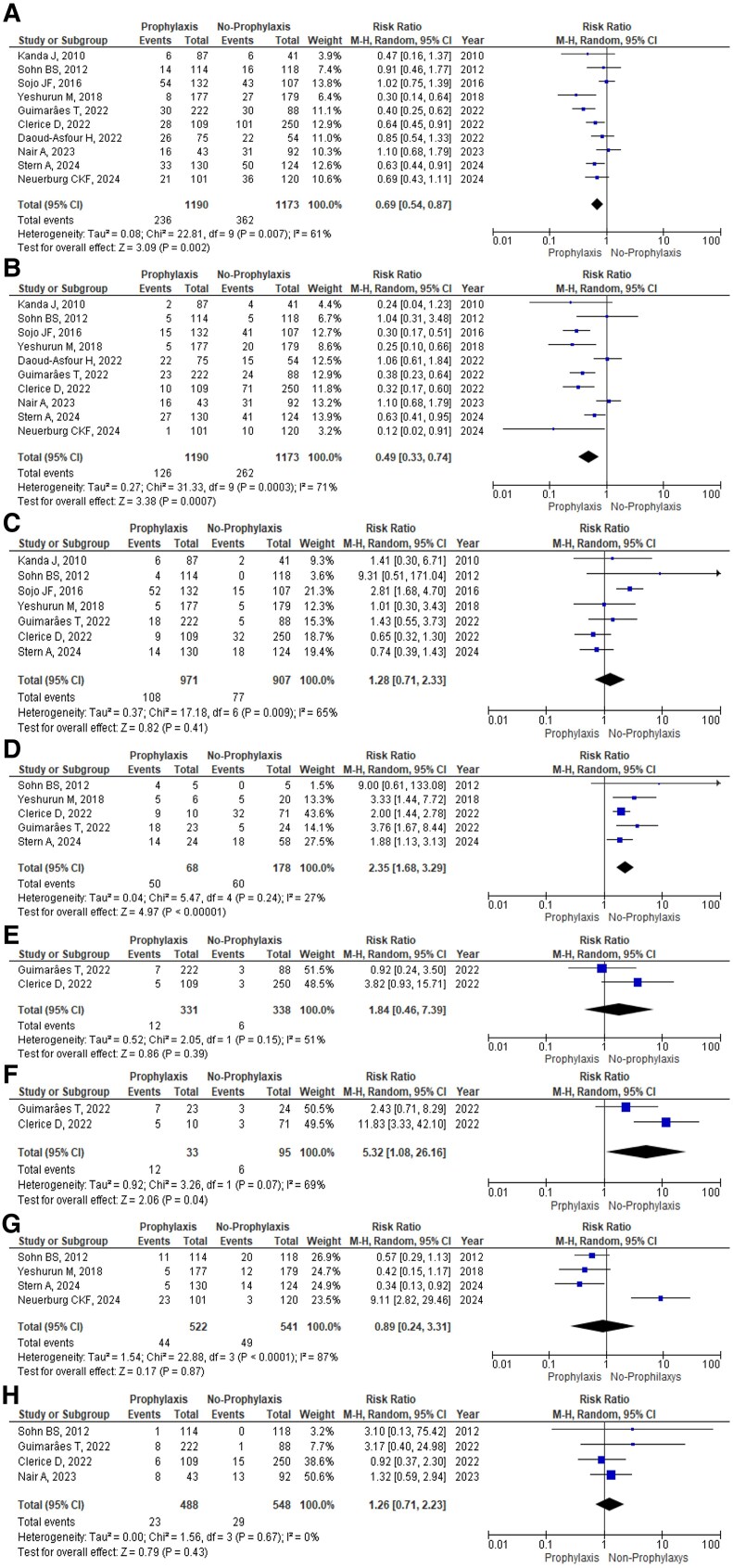
Meta-analysis of the studied outcomes in HSCT recipients before and after pre-engraftment quinolone prophylaxis withdrawn. (*A*) Bloodstream infection by all microorganisms. (*B*) Bloodstream infection by Gram-negative bacilli. (*C*) Infection by quinolone-resistant microorganisms in overall population. (*D*) Infection by quinolone-resistant microorganisms in patients with Gram-negative bloodstream infection. (*E*) Infection by carbapenem-resistant microorganisms in overall population. (*F*) Infection by carbapenem-resistant microorganisms in patients with Gram-negative bloodstream infection. (*G*) *Clostridioides difficile* infection. (*H*) infection-related mortality.

Quinolone prophylaxis was associated with a lower risk of BSIs due to any microorganism (pooled risk ratio: 0.69; 95% CI 0.54–0.87; *P* value = .002; I^2^ = 61%) and due to Gram-negative bacteria (pooled risk ratio: 0.49; 95% CI 0.33–0.74; *P* value = .0007; I^2^ = 71%) (Figures 2*A* and 2*B*). Funnel plots are shown in Supplementary Appendix Figures 4A and 4B). Subgroup analyses according to the type of quinolone used were performed (Supplementary Appendix Figures 5A and 5B).

### Antimicrobial Resistance

Table 3 also presents the prevalence of quinolone-resistant microorganisms and carbapenem-resistant Gram-negative bacteria across the included studies. Seven studies (70.0%) [8, 9, 14–17, 20] were included in the meta-analysis of quinolone-resistance infection in the overall population, and 5 studies (50.0%) [8, 9, 15, 17, 20] in Gram-negative BSI population. Two studies (20.0%) [8, 9] were included in the meta-analysis for carbapenem-resistance.

In the overall population, quinolone prophylaxis was not associated with a statistically significant increase in the risk of infection due to quinolone-resistant microorganisms (pooled risk ratio: 1.28; 95% CI 0.71–2.33; *P* value = .41; I^2^ = 65%) and due to carbapenem-resistant microorganisms (pooled risk ratio: 1.84; 95% CI 0.46–7.39; *P* value = .39; I^2^ = 51%). Among patients who developed Gram-negative BSI, quinolone prophylaxis was associated with a higher risk of infection caused by quinolone-resistant Gram-negative bacteria (pooled risk ratio: 2.35; 95% CI 1.68–3.29; *P* value < .00001; I^2^ = 27%). and carbapenem-resistant Gram-negative bacteria (pooled risk ratio: 5.32; 95% CI 1.08–26.16; *P* value = .04; I^2^ = 69%), although this outcome was reported only in 2 studies [8, 9]. (Figures 2*C*, 2*D*, 2*E* and 2*F*).

Subgroup analyses for infections due to quinolone-resistant microorganisms in the overall population and among patients who developed Gram-negative BSI are presented in Supplementary Appendix Figures 5*C* and 5*D*. Subgroup analysis was not performed for the carbapenem-resistant outcome, as only 2 studies were included. Supplementary Appendix 3 presents the 3rd generation cephalosporin resistance pattern.

### *C. difficile* Infection

Four studies (40.0%) [15, 17, 20, 21] evaluated the prevalence of CDI in both groups, as summarized in Table 3. There was no statistically significant difference between the 2 groups (pooled risk ratio: 0.89; 95% CI 0.24–3.31; *P* value = .87; I^2^ = 87%). (Figure 2*G*).

### Infection-related Mortality

The prevalence of infection-related mortality during follow-up is presented in Table 3, and there was no statistically significant difference observed between the 2 groups (pooled risk ratio: 1.26; 95% CI: 0.71–2.23; *P* value = .43; I^2^ = 0%). (Figure 2*H*). Subgroup analyses for this outcome presented in Supplementary Appendix Figure 5*E*.

## DISCUSSION

This systematic review found 10 studies that evaluated the withdrawal of quinolone prophylaxis in adult HSCT patients. The meta-analysis highlights that discontinuing routine quinolone prophylaxis during pre-engraftment neutropenia in HSCT recipients is associated with an increased incidence of BSIs. However, no statistically significant difference in infection-related mortality was observed. These findings should be interpreted as an initial hypothesis generated from this work, as the available data may be insufficient to exclude clinically meaningful differences in mortality. Moreover, the prevalence of infections with antimicrobial resistance for quinolones and carbapenems reduced significantly after prophylaxis discontinuation.

It has been demonstrated that the use of quinolone prophylaxis in HSCT recipients selects for carbapenem-resistant isolates. Hakki et al reported that breakthrough infections during quinolone prophylaxis were associated with an odds ratio of 14.4 (95% CI: 4.1–62.0; *P* < .001) for carbapenem-resistant *Pseudomonas aeruginosa* BSIs [7]. This observation may be partially explained by the fact that quinolone use promotes the upregulation of efflux pumps (such as MexAB-OprM) [22] and the transcriptional downregulation of OprD, which limits meropenem entry into bacterial cell [7].

HSCT recipients who develop BSIs caused by carbapenem-resistant microorganisms have poor overall survival. In a retrospective cohort of 569 HSCT, 105 (18.4%) were colonized, and 30 (5.3%) developed bloodstream infection (BSI) due to carbapenem-resistant *Klebsiella pneumoniae*. The D + 100 overall survival was 85.8 % in noncolonized patients, 75.4 % in colonized patients, and 35.7 % in infected patients (*P* value < .001) [23]. Outcomes are even worse when HSCT patients develop BSI caused by carbapenem-resistant *P. aeruginosa*, with overall mortality rate reaching 79.0% [24].

The use of antibiotics, including quinolones, has also been associated with a higher risk of acute graft-versus host disease (aGVHD) in allogeneic-HSCT recipients. A recent study involving 2023 allogeneic-HSCT patients showed that quinolone use during week 4 post-transplant was associated with a hazard ratio of 1.82 (95% CI: 1.13–2.93) for grades II-IV of aGVHD, and of 2.70 (95% CI: 1.30–5.59) for grades III-IV [25].

There is growing evidence that antibiotic use, including prophylactic levofloxacin, may have a detrimental effect on the gut microbiome. In an exploratory study, Fabbrini et al [26] evaluated the gut microbiome in 30 pediatric patients undergoing HSCT. The authors found that the combination of parenteral nutrition and levofloxacin prophylaxis was associated with low modularity, poor cohesion, a shift in keystone species and the emergence of modules comprising several pathobionts [26].

In our systematic review, even in the absence of a clear impact on mortality, the observed increase in BSIs could remain clinically relevant, given their association with acute morbidity, increased healthcare costs, and potential long-term complications. For example, severe sepsis during pediatric leukemia treatment has been associated with an increased risk of long-term neurocognitive impairment in adulthood [27]. However, these outcomes were not systematically evaluated in the included studies, potentially leading to an underestimation of the overall clinical burden. Prospective studies with longer follow-up are warranted to better characterize these effects.

Our study has some limitations. The main limitation is the absence of randomized clinical trials. All included studies were observational, with heterogeneous retrospective and prospective designs. Moreover, all studies were single centers. To our knowledge, no randomized clinical trial is currently ongoing to specifically evaluate quinolone prophylaxis discontinuation in HSCT recipients. Another important source of confounding relates to the heterogeneity across transplantation centers and study periods. Conditioning intensity, supportive care practices, and local antimicrobial resistance epidemiology are potential confounders in this context. Unfortunately, these factors were not routinely described in the included cohorts, and therefore their impact could not be assessed. Such heterogeneity limits comparability between studies and could have influenced infectious outcomes and the effect of quinolone prophylaxis. Additionally, none of the included studies reported data on the proportion of patients who continued to receive quinolone prophylaxis after its discontinuation. Residual use of prophylaxis in the postdiscontinuation period could have attenuated the observed effects. Another source of heterogeneity relates to the definition of infection-related mortality across studies. Mortality time points were not uniformly reported, with some studies assessing early mortality and others reporting longer follow-up periods. This discrepancy may have contributed to unmeasured heterogeneity in this outcome. Nevertheless, by evaluating data of 2363 HSCT recipients, this systematic review provides a comprehensive evaluation of the clinical impact of discontinuing quinolone prophylaxis. We were able to demonstrate that this strategy may contribute to reduced prevalence of antimicrobial resistance.

In conclusion, discontinuation of routine quinolone prophylaxis during pre-engraftment neutropenia in HSCT recipients was associated with a predictable increase in BSI. The available data did not demonstrate a statistically significant difference in mortality and prophylaxis discontinuation was not associated with a significant reduction in the risk of resistant infections in the overall population. However, in those who developed Gram-negative BSIs, isolated pathogens were less likely to be quinolone- or carbapenem-resistant. Nevertheless, given the observational design of the available evidence and the presence of unmeasured confounders, these findings should be interpreted as hypothesis-generating rather than definitive and require confirmation in multicenter randomized clinical trials.

## Supplementary Material

ofag358_Supplementary_Data

## Supplementary Data

Supplementary materials are available at *Open Forum Infectious Diseases* online. Consisting of data provided by the authors to benefit the reader, the posted materials are not copyedited and are the sole responsibility of the authors, so questions or comments should be addressed to the corresponding author.
