# Supplementary material for: Effect of Quinolone Prophylaxis Discontinuation During Pre-engraftment Neutropenia on Incidence, Mortality, and Etiology of Bloodstream Infections in Hematopoietic Stem-cell Transplant Recipients: A Systematic Review and Meta-analysis
Source: Open Forum Infect Dis. 2026 Jun 8;13(6):ofag358. doi: 10.1093/ofid/ofag358 (PMC13280638; doi:10.1093/ofid/ofag358)
Supplement: ofag358_Supplementary_Data [file ofag358_supplementary_data.zip › Appendix 1.docx]

**Appendixes**

**Appendix 1:** Strategies and dates of the search in each database

***MEDLINE – date of research:*** Oct-10-2023, updated May-20-2025.

| **Strategy** |
| --- |
| ("Hematopoietic Stem Cell Transplantation"[Mesh]) OR ("Stem Cell transplantation"[Mesh]) OR ("Peripheral blood stem cell transplantation"[Mesh]) OR ("Transplantation, Autologous"[Mesh]) OR ("Transplantation, Haploidentical"[Mesh]) OR (Bone Marrow/transplantation) OR (Hematopoietic Stem Cells/transplantation) OR ("Bone marrow transplantation") OR ("Allogeneic Transplantation") OR ("Transplantation, Allogeneic") OR ("Haploidentical Transplantations") OR (Transplantations, Haploidentical) OR ("Haploidentical Transplantation") OR ("Stem Cell Transplantation, Hematopoietic") OR ("Transplantation, Hematopoietic Stem Cell") OR ("Autologous Transplantation") OR ("Autologous Transplantations") OR (Haematopoietic stem cell transplant*) OR (Haematopoietic stem cell-transplant*) OR (Hematopoietic Stem-cell transplant*) AND (quinolone OR ciprofloxacin OR levofloxacin OR quinolones[Mesh]) AND (prophylaxis OR "antibiotic prophylaxis"[Mesh]) |

***EMBASE – date of research:*** Oct-10-2023, updated May-20-2025.

| **Strategy** |
| --- |
| ('hematopoietic stem cell transplantation'/exp OR 'bone marrow transplantation'/exp) AND ('quinolone'/exp OR 'ciprofloxacin'/exp OR 'levofloxacin'/exp) AND ('prophylaxis'/exp) |

***SCOPUS – date of research:*** Oct-10-2023, updated May-20-2025.

| **Strategy** |
| --- |
| (hematopoietic AND stem AND cell AND transplantation OR hsct OR hematopoietic AND stem-cell AND transplantation OR bone AND marrow AND transplantation OR bmt) AND (quinolone OR ciprofloxacin OR levofloxacin) AND prophylaxis |

***LILACS – date of research:*** Oct-10-2023, updated May-20-2025.

| **Strategy** |
| --- |
| (hematopoietic stem cell transplantation OR HSCT OR transplante de medula) AND (quinolone OR ciprofloxacino OR ciprofloxacina OR levofloxacino OR levofloxacina) AND (prophylaxis OR profilaxia) |

***Web of Science – date of research:*** Oct-10-2023, updated May-20-2025.

| **Strategy** |
| --- |
| (hematopoietic stem cell transplantation OR HSCT or bone marrow transplantation OR BMT or hematopoietic stem-cell transplantation) AND (quinolone OR ciprofloxacin OR levofloxacin) AND (prophylaxis) |
