# Supplementary material for: Effect of Quinolone Prophylaxis Discontinuation During Pre-engraftment Neutropenia on Incidence, Mortality, and Etiology of Bloodstream Infections in Hematopoietic Stem-cell Transplant Recipients: A Systematic Review and Meta-analysis
Source: Open Forum Infect Dis. 2026 Jun 8;13(6):ofag358. doi: 10.1093/ofid/ofag358 (PMC13280638; doi:10.1093/ofid/ofag358)
Supplement: ofag358_Supplementary_Data [file ofag358_supplementary_data.zip › Appendix 2.docx]

**Appendix 2:** Quality assessment of included studies using the JBI Critical Appraisal Checklist

| **Reference, Year** | Is it clear in the study what is the “cause” and what is the “effect” (i.e. there is no confusion about which variable comes first)? | Were the participants included in any comparisons similar? | Were the participants included in any comparisons receiving similar treatment/care, other than the exposure or intervention of interest? | Was there a control group? | Were there multiple measurements of the outcome both pre and post the intervention/exposure? | Was follow up complete and if not, were differences between groups in terms of their follow up adequately described and analyzed? | Were the outcomes of participants included in any comparisons measured in the same way? | Were outcomes measured in a reliable way? | Was appropriate statistical analysis used? | **Risk of Bias** |
| --- | --- | --- | --- | --- | --- | --- | --- | --- | --- | --- |
| Kanda J, 2010 | Y | Y | Y | N | Y | Y | Y | N | Y | Moderate |
| Sohn BS, 2012 | Y | Y | Y | N | Y | Y | Y | Y | Y | Low |
| Sojo JF, 2016 | Y | Y | Y | N | Y | Y | Y | N | Y | Moderate |
| Yeshurun M, 2018 | Y | Y | Y | N | Y | Y | Y | N | Y | Moderate |
| Daoud-Asfour H, 2022 | Y | Y | Y | N | Y | Y | Y | N | Y | Moderate |
| Guimarães T, 2022 | Y | Y | Y | N | Y | Y | Y | Y | Y | Low |
| Clerici D, 2022 | Y | Y | Y | N | Y | Y | Y | Y | Y | Low |
| Nair A, 2023 | Y | Y | Y | N | Y | Y | Y | Y | Y | Low |
| Stern A, 2024 | Y | Y | Y | N | Y | Y | Y | N | Y | Moderate |
| Neuerburg CKF, 2024 | Y | Y | Y | N | Y | Y | Y | N | Y | Moderate |

Y: Yes; N: No; NA: not applicable; U: Unclear
