# Supplementary material for: Effect of Quinolone Prophylaxis Discontinuation During Pre-engraftment Neutropenia on Incidence, Mortality, and Etiology of Bloodstream Infections in Hematopoietic Stem-cell Transplant Recipients: A Systematic Review and Meta-analysis
Source: Open Forum Infect Dis. 2026 Jun 8;13(6):ofag358. doi: 10.1093/ofid/ofag358 (PMC13280638; doi:10.1093/ofid/ofag358)
Supplement: ofag358_Supplementary_Data [file ofag358_supplementary_data.zip › Appendix 3.docx]

**Appendix 3**: Prevalence of blood cultures with Enterobacteriales or Non-fermentative Gram-negative bacteria and 3^rd^ generation cephalosporin resistance: effect of quinolone prophylaxis discontinuation in HSCT patients

| **Reference, Year** | **Enterobacteriales isolated in blood cultures** | | | **Non-fermentative isolated in blood cultures** | | | **3^rd^ generation cephalosporin-resistant GN** | | |
| --- | --- | --- | --- | --- | --- | --- | --- | --- | --- |
|  | **Prophylaxis**  **N (%)** | **No prophylaxis**  **N (%)** | ***p*-value** | **Prophylaxis**  **N (%)** | **No prophylaxis**  **N (%)** | ***p*-value** | **Prophylaxis**  **N (%)** | **No prophylaxis**  **N (%)** | ***p*-value** |
| Kanda J, 2010 | NA | NA | NA | NA | NA | NA | NA | NA | NA |
| Sohn BS, 2012 | 5 (35.7) | 2 (12.5) | NA | 0 | 1 (0.8) | 0.26 | 1 (0.8) | 0 | NA |
| Sojo JF, 2016 | NA | NA | NA | NA | NA | NA | NA | NA | NA |
| Yeshurun M, 2018 | NA | NA | NA | NA | NA | NA | NA | Na | NA |
| Daoud-Asfour H, 2022 | 20 (67.0) | 22 (73.0) | 0.58 | 2 (7.0) | 2 (7.0) | 1.00 | 13 (43.0) | 4 (13.0) | 0.02 |
| Guimarães T, 2022 | 30 (85.7) | 16 (84.2) | NA | 3 (8.57) | 2 (10.5) | NA | 10 (30.3) | 2 (11.1) | NA |
| Clerici D, 2022 | 8 (7.3) | 56 (22.4) | NA | 2 (1.8) | 10 (4.0) | NA | NA | NA | NA |
| Nair A, 2023 | NA | NA | NA | NA | NA | NA | NA | NA | NA |
| Stern A, 2024 | 17 (34.1) | 46 (58.2) | NA | 8 (10.1) | 12 (15.1) | NA | NA | NA | NA |
| Neuerburg CKF, 2024 | 1 (1.0) | 8 (6.7) | NA | 0 | 2 (1.7) | NA | NA | NA | NA |

BSI: bloodstream infection; GN: Gram-negative; NA: not available
