# Supplementary material for: Effect of Quinolone Prophylaxis Discontinuation During Pre-engraftment Neutropenia on Incidence, Mortality, and Etiology of Bloodstream Infections in Hematopoietic Stem-cell Transplant Recipients: A Systematic Review and Meta-analysis
Source: Open Forum Infect Dis. 2026 Jun 8;13(6):ofag358. doi: 10.1093/ofid/ofag358 (PMC13280638; doi:10.1093/ofid/ofag358)
Supplement: ofag358_Supplementary_Data [file ofag358_supplementary_data.zip › Figure legends.docx]

**Figure 1:** Flowchart of the selection of the studies included in the systematic review

**Figure 2:** Meta-analysis of the studied outcomes in HSCT recipients before and after pre-engraftment quinolone prophylaxis withdrawn. A: bloodstream infection by all microorganisms. B: bloodstream infection by Gram-negative bacilli. C: infection by quinolone-resistant microorganisms in overall population. D: infection by quinolone-resistant microorganisms in patients with Gram-negative bloodstream infection. E: infection by carbapenem-resistant microorganisms in overall population. F: infection by carbapenem-resistant microorganisms in patients with Gram-negative bloodstream infection. G: *Clostridioides difficile* infection. H: infection-related mortality.

**Appendix 4:** Funnel plot for the meta-analysis with ten or more included studies. A: bloodstream infection by all microorganisms. B: bloodstream infection by Gram-negative bacilli.

**Appendix 5:** Subgroup analyses by quinolone type (levofloxacin vs ciprofloxacin) for study outcomes. A: bloodstream infection by all microorganisms. B: bloodstream infection by Gram-negative bacilli. C: infection by quinolone-resistant microorganisms in overall population. D: infection by quinolone-resistant microorganisms in patients with Gram-negative bloodstream infection. E: infection-related mortality.
