# Supplementary figures and images for: Effect of Quinolone Prophylaxis Discontinuation During Pre-engraftment Neutropenia on Incidence, Mortality, and Etiology of Bloodstream Infections in Hematopoietic Stem-cell Transplant Recipients: A Systematic Review and Meta-analysis
Source: Open Forum Infect Dis. 2026 Jun 8;13(6):ofag358. doi: 10.1093/ofid/ofag358 (PMC13280638; doi:10.1093/ofid/ofag358)

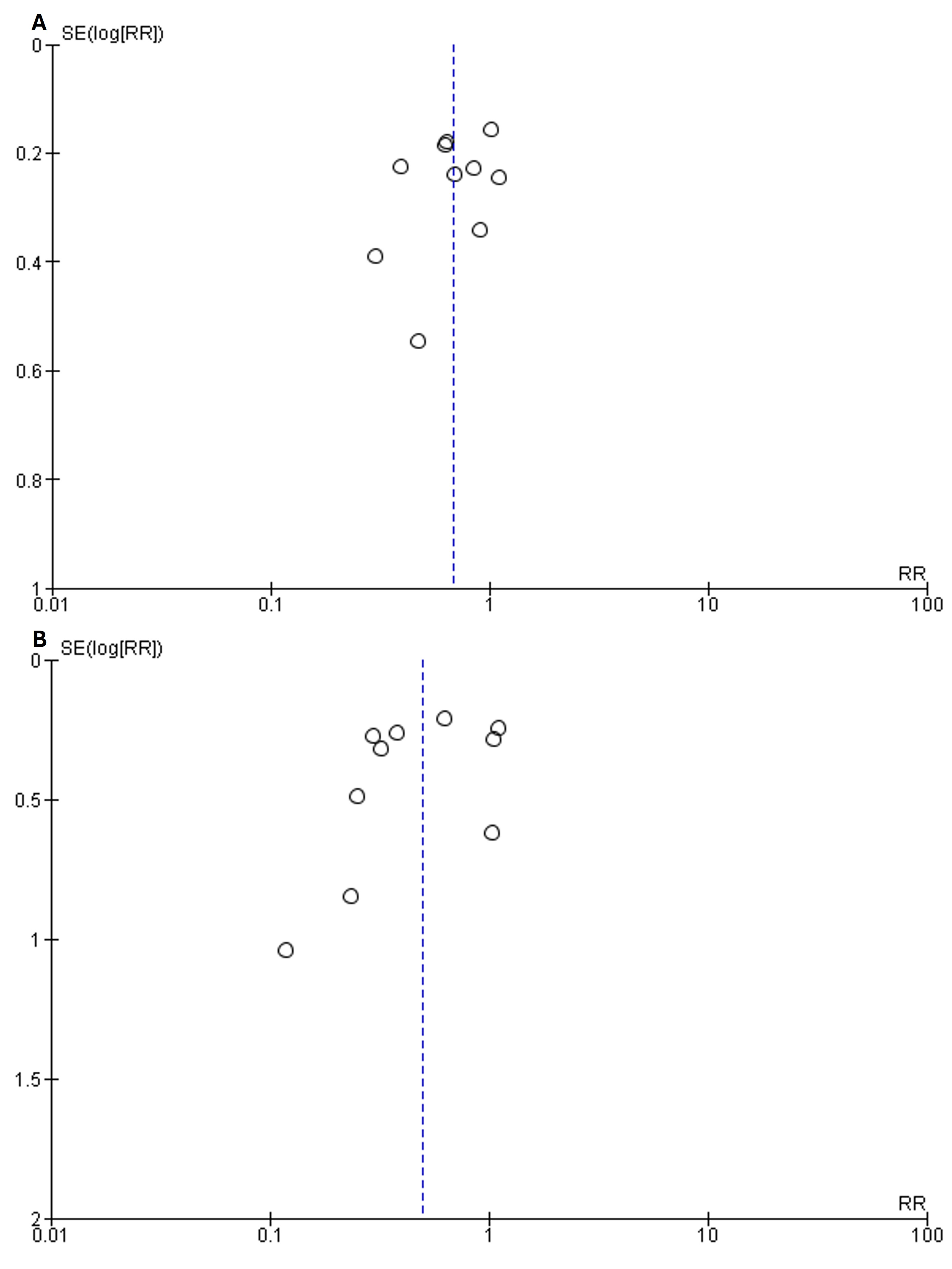

Supplement: ofag358_Supplementary_Data [file ofag358_supplementary_data.zip › Appendix 4.tiff]

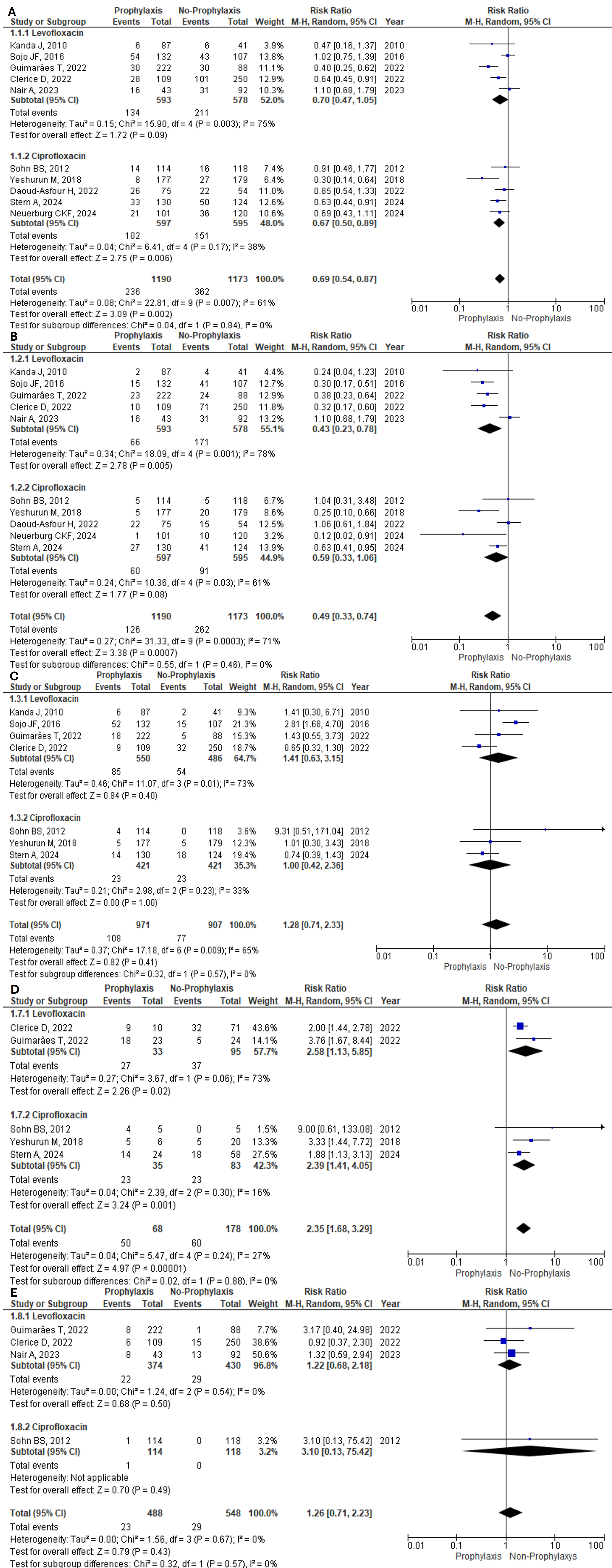

Supplement: ofag358_Supplementary_Data [file ofag358_supplementary_data.zip › Appendix 5.tiff]
